# Evaluating the impact of the national health insurance scheme of Ghana on out of pocket expenditures: a systematic review

**DOI:** 10.1186/s12913-018-3249-9

**Published:** 2018-06-07

**Authors:** Juliet Okoroh, Samuel Essoun, Anthony Seddoh, Hobart Harris, Joel S. Weissman, Lydia Dsane-Selby, Robert Riviello

**Affiliations:** 1 Center for Surgery and Public Health, Brigham and Women’s Hospital, Boston, MA USA; 2 Department of Surgery, Korle-Bu Teaching Hospital, Accra, Ghana; 3 World Bank Working Group, Accra, Ghana; 4 Department of Surgery, University of California San Francisco, 513 Parnassus Ave S-321, San Francisco, CA 94143 USA; 5 National Health Insurance Authority, Accra, Ghana; 6 Fogarty International Center, National Institute of Health, GloCal Consortium, Bethesda, USA

**Keywords:** Universal health coverage, Health systems strengthening, National health insurance schemes, Out of pocket payments for health in sub-Saharan Africa (SSA), Catastrophic health expenditures

## Abstract

**Background:**

Approximately 150 million people suffer from financial catastrophe annually because of out-of-pocket expenditures (OOPEs) on health. Although the National Health Insurance Scheme (NHIS) of Ghana was designed to promote universal health coverage, OOPEs as a proportion of total health expenditures remains elevated at 26%, exceeding the WHO’s recommendations of less than 15–20%. To determine whether enrollment in the NHIS reduces the likelihood of OOPEs and catastrophic health expenditures (CHEs) in Ghana, we undertook a systematic review of the published literature.

**Methods:**

We searched for quantitative articles published in English between January 1, 2003 and August 22, 2017 in PubMed, Google Scholar, Economic Literature, Global Health, PAIS International, and African Index Medicus. Two independent authors (J.S.O. & S.E.) reviewed the articles for inclusion, extracted the data, and conducted a quality assessment of the studies. We accepted the World Health Organization definition of catastrophic health expenditures which is out of pocket payments for health care which exceeds 20% of annual house hold income, 10% of household expenditures, or 40% of subsistence expenditures (total household expenditures net food expenditures).

**Results:**

Of the 1094 articles initially identified, 7 were eligible for inclusion. These were cross-sectional household studies published between 2008 and 2016 in Ghana. They demonstrated that the uninsured paid 1.4 to 10 times more in out-of-pocket payments (OOPs) and were more likely to incur CHEs than the insured. Yet, 6 to 18% of insured households made catastrophic payments for healthcare and all studies reported insured members making OOPs for medicines.

**Conclusion:**

Evidence suggests that the national health insurance scheme of Ghana over the last 14 years has made some impact on reducing OOPEs, and yet healthcare costs remain catastrophic for a large proportion of insured households in Ghana. Future studies need to explore reasons for the persistence of OOPs for medicines and services that are covered under the scheme.

**Electronic supplementary material:**

The online version of this article (10.1186/s12913-018-3249-9) contains supplementary material, which is available to authorized users.

## Background

It is estimated that each year, approximately 150 million people suffer financial catastrophe, spending greater than 40% of non-food expenditures on health, and 100 million people are pushed under the poverty limit because of out-of-pocket spending on health [1–3]. In fact, 5.6 billion people in low and middle income countries (LMICs) depend on out-of-pocket payments (OOPs) to cover more than 50% of their health care expenditures [4]. The implications of this spending in the context of extreme poverty, social development, and human rights are far reaching. In 2011, the World Health Assembly passed Resolution 64.9, recognizing the need for health systems to be equitable and safe, with an action plan to accomplish this through universal health coverage [5].

Ghana is an LMIC in the heart of West Africa with a population of 27 million people and was the first country in Africa to gain its independence in 1957 from British colonial rule. Before 2003, Ghana’s healthcare financing was through “cash and carry”, with out of pocket expenditures (OOPEs) accounting for close to 50% of total health expenditures in the country [6]. There were few community health insurance schemes but healthcare was largely unaffordable for the poor [7]. Ghana renewed its commitment to Universal Health Coverage in 2003 through the passage of the National Health Insurance Scheme (NHIS) Act 650, the purpose of which was to “*ensure equitable and universal access for all residents of Ghana to an acceptable quality package of essential healthcare”* [8]. Furthermore, every resident of Ghana was to belong to a health insurance scheme that *“adequately covers… against the need to pay out of pocket at the point of service use”,* [8] making Ghana one of the first countries in Sub-Saharan Africa to propose an NHIS [9].

The NHIS in Ghana is a government-sponsored social health insurance scheme funded primarily by taxation through the National Health Insurance Levy (70%), social security contributions (17%), investment income (8%), and premiums and registration fees (5%). It covers 95% of health conditions in Ghana and includes access to a variety of inpatient and outpatient services [10]. It also includes access to surgical care, emergency care, and obstetrics. To enroll, individuals have to pay a registration fee and annual premium between 7.2 Ghanaian Cedis (GH¢) and 48 GH¢ ($2.0–$10 USD), which is based on income and ability to pay [11]. As of 2013, approximately 40% of the population was actively enrolled in the scheme, with ongoing plans by the government to increase enrollment, particularly for the poor [12].

### Equity and financial inclusivity of NHIS

Equity and financial inclusion have been part of the core mission of the NHIS since its inception in 2003. As the law stands, pregnant women, children under the age of 18, people living with mental and physical disabilities, poor indigenes, and people over the age of 70 are excluded from premium payments. The overall scheme was designed to be progressive and equitable: more than 60% of the subscribed population is exempt from paying premiums and there are no copayments at the point of care [12]. In reality, there are many challenges with the identification and enrollment of the poor for whom OOPEs present a greater threat of catastrophic expenses. A study of 5500 households in the poorest districts in Northern Ghana found that only 33% of respondents in the poorest quintile were insured compared to 58% of respondents in the richest quintile [13]. A study of 7000 individuals in the Central and Eastern region of Ghana found that despite knowledge of the scheme and its benefits, only 17% of the poorest were insured compared to 44% of the richest households [14]. Furthermore, inability to afford the premiums, perceptions of good health, and poor service quality have been cited amongst other reasons why some people remain uninsured [15–18]. In contrast, a 2015 study of 2500 households found that despite 64% of the uninsured households reporting the cost of the premiums and registration fees as a reason for not enrolling, 70% of these households could afford the premiums, which accounted for less than 2% of their annual household expenditures [11]. This finding is consistent with another study which showed that only 1.5% of the poor are at risk of catastrophic payments and proposed that some premium exempt members could contribute to the scheme [19].

Regardless of the affordability of the premiums, enrolled individuals still make OOPEs at the point of care in the form of user fees, consultation fees, and payments for medicines that are covered under the scheme. This type of spending goes against the NHIS core mission to.

“*adequately covers against the need to pay out of pocket at the point of service”* [8]*.* The OOPEs put poorer households at financial risk as they are more likely to forego care, borrow, or liquidate assets in order to afford needed health services [20]. In fact, OOPEs as a percent of total health expenditures in Ghana are at 26%, which exceeds the WHO recommendations of less than 15–20% and is considered catastrophic [21, 22]. In this systematic review, we aimed to summarize the evidence on whether enrollment in the NHIS makes a difference in OOPEs, and a difference on catastrophic health expenditures (CHEs) in Ghana.

## Methods

### Search strategy and selection criteria

We did a systematic review using the Preferred Reporting Items for Systematic Reviews and Meta-Analysis (PRISMA) guidelines [23]. We searched PubMed, Google Scholar, Economic Literature, Global Health, PAIS International, and African Index Medicus for articles published from January 1, 2003 to August 22, 2017, using predefined search criteria and the following search strategy based on the following Medical Subject Headings (MeSH) terms for insurance: “insurance or national health programs or insured or uninsured or national health or catastrophic health or universal health or universal coverage or health coverage”. This was combined with MeSH terms for the country: “Ghana or Ghanaian” using the conjunction “AND”*.* The same search was conducted in all seven databases. A comprehensive list of MeSH terms used to identify quantitative studies on the impact of insurance OOPEs and financial catastrophe is available in Additional file 1.

### Criteria for full-text review

We included all studies published since the NHIS was enacted in 2003. Studies whose primary outcome was not financial catastrophe but provided a secondary analysis with comparisons of OOPEs/financial catastrophe by insurance status were also included. Both prospective and retrospective studies were considered for inclusion. Authors were contacted in the event that their articles were not available for full-text review and considered if made available. Using PICO’s framework (Population, Intervention, Comparison, Outcome) for the literature search, our sampling population was defined as studies on individuals or households in Ghana [24]. The intervention being examined was insurance status as defined by enrollment in the NHIS or not. The comparison group was the uninsured. Our primary outcome was defined as OOPEs, which included direct costs, and indirect costs such as transportation costs, and lost productivity or wages. The secondary outcome of interest was CHEs, defined by the WHO as health expenditures that exceed 10% of total household expenditures, 20% of total household income, or 40% of non-food expenditures. The OOPE(s) is defined from the patient’s perspective: i.e. payments made by individuals or households, to health facilities which was not reimbursed by the health insurance scheme.

We excluded studies based on their titles and abstract if deemed not relevant to the topic, and studies not on the Ghanaian population and not published in English. In the event a study focused on the differences in health insurance coverage in select countries in Sub-Saharan Africa (SSA), only the analysis on Ghana was included. Because we were interested in quantitative studies only, we excluded technical notes, case reports, and literature reviews. Focus groups, stakeholder analyses, studies on adverse selection, and moral hazard under the NHIS were excluded. Studies with no clear aims or objectives, no statistical analysis, or that were not peer-reviewed were also excluded. Articles that discussed differences in OOPEs or financial catastrophe by insurance status in the text, but without stratification in tables and statistical analysis were also excluded.

### Data analysis

Data collection was completed by two independent reviewers (J.S.O, S.E) using a standard data extraction form (Table 1). We collected general information about the articles: the study citation, authorship, year, and type of publication. Study characteristics included study objectives, design, data source, sampling technique, power calculation, and study setting. We collected information regarding the participant characteristics, including a description of the study population, control groups, inclusion of socioeconomic status (SES) in the analysis, and population size. Outcomes were types of costs measured (direct, indirect such as transportation, and lost wages/productivity). We examined the inclusion of financial risk protection in the analysis and how this was measured. Whether the articles reported a reduction in OOPEs, CHEs, or poverty reduction was also examined. For the statistical analysis, we collected the odds ratios, *P*-values, confidence intervals, and the definitions of the variables used in the authors’ analysis that compared differences between the insured and uninsured.

**Table 1:** Data Extraction Form

General Information	
Initials of the reviewer	
Date the review was conducted	
Citation/Title	
Journal/publication body	
Publication year	
PubMed ID (for referencing only)	
Study Characteristics	
Objectives of the study	
Study design	
Data source	
Sampling technique	
Justification of the sample size	
Power calculation	
Study setting	
Participant Characteristics	
Description of the study population	
Population size	
Description of the control group	
Inclusion of socio-economic classification	
Description of socio-economic status (variables included) in the analysis	
Outcomes Measured	
Types of health-care costs measured in the studies (direct and indirect costs such as transportation cost and lost wages)	
Measures of financial protection used in the analysis	
Reported differences in out of pocket expenditures by insurance status	
Reported differences in catastrophic health expenditures by insurance status	
Any report of poverty reduction by insurance status	
Type of statistical analysis used by the authors	
Key findings of the studies	
Discussion of generalizability	

To assess the quality of the articles meeting the inclusion criteria, the two reviewers used a checklist similar to that used by Mirza and Jenkins [25]. The checklist included eight quality items: 1) Explicit study aims stated; 2) Sample size justification given; 3) Representative sample or justification; 4) Clear inclusion and exclusion criteria; 5) Reliability and validity of measures justified; 6) Adequate description of the data; 7) Statistical significance assessed; 8) Discussion on the generalizability of the study provided. One point was given for a “yes” answer and none for a “no” answer, for a possible maximum score of eight points (Table 2). We could not assess the methodological quality of the authors’ data collection and source; that is, how information on insurance status, costs of care, household expenditures, and household income were collected. We simply reported this information when made available. Because of the scarcity of available literature on this topic, we included grey literature and assigned high, moderate, or low quality to studies based on strength of the outcomes reported, in addition to the total scores by the two reviewers. Articles that reported differences in OOPEs, or CHEs, or poverty reduction by insurance status using WHO standards was rated “high” in quality.

**Table 2:** Methodological quality of studies on the impact of Ghana’s national health insurance scheme on OOPE(s) and financial catastrophe (1 = Yes; 0 = No)

Study	Clear study aims	Adequate sample size(justification)	Representative sample(with justification)	Clear inclusion & exclusion criteria	Reliability & validity of measures justified	Adequate description of the data	Appropriate statistical analysis	Discussion of generalizability	^a^ Total score (J.S.O, S.E)	^a^ Quality based on total score (J.S.O)	^a^ Quality based on total score (S.E)	^b^ Quality based on outcomes
Chankova et al., 2008	1	0	0	0	0	1	1	1	4	Low	Low	Low
Nguyen et al., 2011	0	1	1	1	1	1	1	1	7	High	High	High
Dalaba et al., 2014	1	1	1	1	0	1	0.5	0.5	5.5	Low	Low	Low
Abrokwah et al., 2014	0.5	0.5	1	1	0	1	1	0.5	5.5	Moderate	Moderate	Moderate
Abuosi et al., 2015	1	1	1	1	0	1	1	0	6	Moderate	Low	Low
Kusi et al., 2015	1	1	1	1	1	1	1	1	8	High	High	High
Aryeetey et al., 2016	1	1	1	1	1	1	1	1	8	High	High	High

^a^The total score is the average score for the quality items based on each author’s review. Scores less than 5 were considered low, 5–6 moderate, 7–8 high in quality

^b^Both authors agreed on the quality assignments based on the outcomes reported on OOPE, CHE, or poverty reduction

We could not adequately assess the risk of publication bias or selective reporting because all studies were cross-sectional with minimal adjustments for confounders, and very few studies examined the impact of medical comorbidities, socioeconomic status, moral hazard, adverse selection, and overall cost of caring for sicker patients, which could contribute to differences in OOPEs by insurance status. We summarized the key findings of the articles and any discussion on the generalizability of the studies by the authors. Both reviewers agreed on 87% of the studies included (k score was 0.80). Because of the methodological and statistical heterogeneity between the studies a meta-analysis was not performed. We present the findings of the articles as they relate to our study aim, which was to examine the impact of the NHIS on OOPEs and CHEs in Ghana.

## Results

**Fig. 1:**
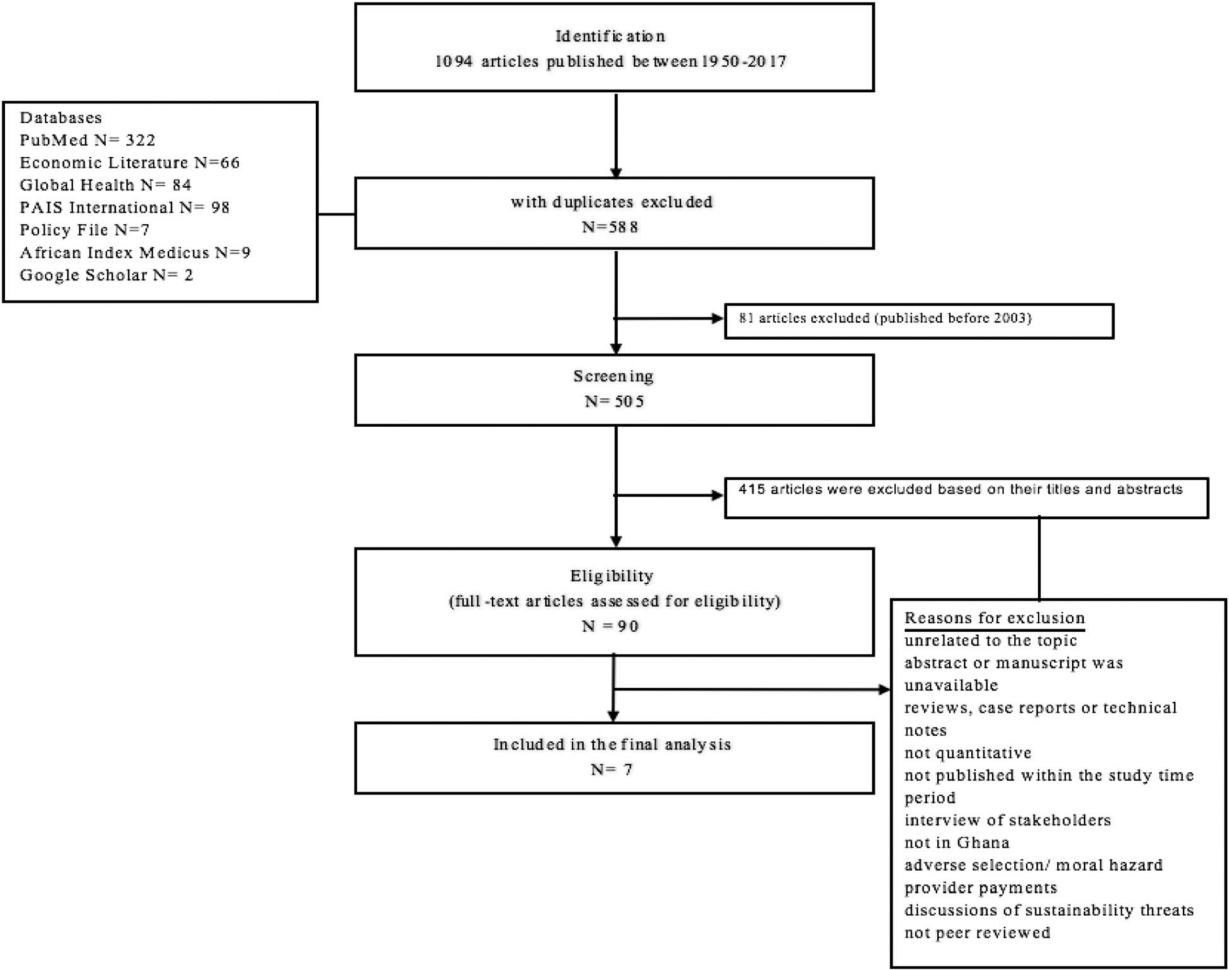
PRISMA flowchart of study selection

A total of 1094 articles were initially identified; 588 articles with duplicates were excluded, 81 because they were published before the NHIS was enacted in 2003, and 415 on the basis of their titles and abstract (Fig. 1). Ninety articles were eligible for full text review, seven of which met our inclusion criteria. Table 2 summarizes the results of the quality assessment of the studies conducted by the two independent reviewers (J.S.O and S.E). Tables 3 and 4 provide a general description of the studies included: study aims/objectives, study population and size, study design, setting, statistical analysis, and key findings.

**Table 3:** Summary of survey methodology and participant characteristics in the included studies

	General Information			Study Characteristics					Participant Characteristics		
First Author	Publication Year	Citation/Title	Study Objectives/Aims	Study Design	Data Source	Sampling Technique	Recall Period	Setting^a^	Study Population	SES^b^	Population Size
Chankova	2008	Chankova S, Sulzbach S, Diop F. Impact of mutual health organizations: evidence from West Africa. Health Policy and Planning. 2008;23(4):264–276.	To answer the following questions: (1) Do MHOs include vulnerable populations. (2) Do they have an impact on the utilization of curative services. (3) On out-of-pocket expenditures	Cross-sectional	1°	NR^c^	Twelve months	Household	Three country comparisons; Ghana, Mali, Senegal. (Nkoranza and Offinso districts in Ghana)	SES wealth quintiles, household head, education, occupation, residence(urban-rural), house-hold size	1806 households (34% NHIS, 66% uninsured)
Nguyen	2011	Nguyen HT, Rajkotia Y, Wang H. The financial protection effect of Ghana National Health Insurance Scheme: evidence from a study in two rural districts. International Journal for Equity in Health. 2011, 10: 4–10.1186/1475-9276-10-4.	Not clearly stated but evaluated the impact of NHIS on health service utilization and OOPE(s)	Cross-sectional	1°	Two-stage cluster & random sampling	Two weeks (injury recall period) to twelve months	Household	Households in two districts (Offinso and Nkoranza) in Ghana	SES wealth quintiles, household head, employment status, house-hold size, ethnicity, residence(urban-rural)	11,617 individuals (35% NHIS, 65% uninsured)
Dalaba	2014	Dalaba M, Akweongo P, Aborigo R, Awine T, Azongo D, Asaana P et al. Does the national health insurance scheme in Ghana reduce household cost of treating malaria in the Kassena-Nankana districts? Global Health Action. 2014;7(1):23848.	To examine the effect of NHIS in reducing household cost of treating malaria	Cross-sectional	1°	Convenience random sampling	NR^c^	Household	Households in the Kassena-Nankana district	SES wealth quintiles, age, occupation	4226 households (49.1% NHIS, 50.9% uninsured)
Abrokwah	2014	Abrokwah SO, Moser CM, Norton EC. The effect of social health insurance on prenatal care: the case of Ghana. Int J Health Care Finance Econ. 2014;14(4):385–406.	To describe how Ghana’s health insurance scheme affects prenatal care and out-of-pocket expenditures	Cross-sectional	2° GLS5 2005–2006	Random stratified sampling	Twelve months	Household	Women of child bearing age (15–49 years)	SES wealth quintiles, age, education, region, marital status, occupation, employment status, house-hold size	1032 women from the GLS5 (36% NHIS, 64% uninsured)
**Abuosi**	2015	Abuosi A, Adzei F, Anarfi J, Badasu D, Atobrah D, Yawson A. Investigating parents/caregivers financial burden of care for children with non-communicable diseases in Ghana. BMC Pediatrics. 2015;15(1).	To assess the extent to which parents/caregivers of children with NCDs experience financial burden in caring for them	Cross-sectional	1°	Convenience random sampling	NR^c^	Inpatient	Parents/caregivers of children hospitalized with NCDs at hospitals in Greater Accra, Ashanti, and the Volta region	Parents’ age, education, income, marital status, religion, residence (urban-rural)	225 parents/caregivers (87% NHIS 13% uninsured)
Kusi	2015	Kusi A, Hansen K, Asante F, Enemark U. Does the National Health Insurance Scheme provide financial protection to households in Ghana? BMC Health Services Research. 2015;15(1).	To assess the effect of NHIS on household OOPE(s) and CHE(s)	Cross-sectional	1°	Random stratified Sampling	Four weeks	Household	Households in three districts in the three ecological zones of Southern (Kwaebibrirem), Middle (Asutifi), and Northern (Savelugu-Nanton)	SES wealth quintiles household size, household head, marital status, residence (urban-rural), education, distance to the nearest facility, mode of transportation	2430 households (28% NHIS, 46% uninsured, & 26% partially insured)
Aryeetey	2016	Aryeetey G, Westeneng J, Spaan E, Jehu-Appiah C, Agyepong I, Baltussen R. Can health insurance protect against out-of-pocket and catastrophic expenditures and also support poverty reduction? Evidence from Ghana’s National Health Insurance Scheme. International Journal for Equity in Health. 2016;15(1).	To examine whether Ghana’s health insurance scheme reduces OOPE(s), CHE(s) and poverty at the household level	Cross-sectional	1°	Random stratified sampling	Four weeks	Household	Households in the Eastern and Central Region. Baseline study conducted in 2009 and follow-up in 2011	Household size, marital status, religion, education, residence (urban-rural), occupation, household income, household expenditures	In 2009, 3300 households (31% NHIS 69% uninsured); 2011 3152 households (38% NHIS 62% uninsured)

1° denotes primary data collection by the authors. 2° is secondary analysis of previously collected data. ^a^ Study setting denotes where participants were interviewed

^b^SES wealth quintile refers to the reporting of wealth-specific results using a principal component analysis of dwelling characteristics, access to utilities and ownership of house-hold items. Further description is available at https://www.dhsprogram.com/topics/wealth-index/Wealth-Index-Construction.cfm

^c^NR not reported by the studies

**Table 4:** Summary of Statistical Analysis and Outcomes Reported by the Included Studies

General Information			Outcomes					Statistical Analysis		
First Author	Publication Year	Citation/Title	Outcomes measured	Measures of financial risk protection	Reduction in out of pocket expenditure (OOPE)	Reduction in catastrophic health expenditure (CHE)	Poverty reduction	Type of statistical analysis	Logistic regression (N) number of variables	Key findings
*Chankova*	2008	Chankova S, Sulzbach S, Diop F. Impact of mutual health organizations: evidence from West Africa. Health Policy and Planning. 2008;23(4):264–276.	Direct OOPE(s) for inpatient, outpatient care, & transportation cost	NR^1^	OPD^3^ NS^2^ Transportation NS^2^ IPD OOPE*** (NHIS $4.25USD, uninsured $43.88USD)	NR^1^	NR^1^	Descriptive statistics, logistic regression	(8) Independent variables, dependent variable (OOPE)	1.) Insurance was associated with lower out of pocket payments for inpatient care. 2.) No significance difference in outpatient care. 3.) No difference in transportation cost
*Nguyen*	2011	Nguyen HT, Rajkotia Y, Wang H. The financial protection effect of Ghana National Health Insurance Scheme: evidence from a study in two rural districts. International Journal for Equity in Health. 2011, 10: 4–10.1186/1475-9276-10-4.	OOPE(s) & CHE(s) for illness, surgery, ANC & inpatient care	4 indicators of CHE(s); (5% & 10% of individual income) and (10% & 20% of SE(s)^5^	OOPE*** NHIS 21000 GH¢ ($2.3 USD), uninsured 30,000 GH¢ ($ 3.2 USD)	NHIS reduced CHE(s) by 0.5 to 1% depending on the threshold used.	NR^1^	Descriptive statistics, logistic regression	(6) Independent variables, dependent variable (OOPE)	NHIS reduces the probability of incurring CHE(s).
*Dalaba*	2014	Dalaba M, Akweongo P, Aborigo R, Awine T, Azongo D, Asaana P et al. Does the national health insurance scheme in Ghana reduce household cost of treating malaria in the Kassena-Nankana districts? Global Health Action. 2014;7(1):23848.	Direct OOPE(s) for malaria treatment, lost wages & transportation cost	NR^1^	NS^2^	NR^1^	NR^1^	Descriptive statistics	NR^1^	1.) NHIS has some protective effect on cost of malaria treatment, however not statistically significant 2.) Indirect costs of treating malaria were three times higher than direct costs for both insured and uninsured households.
*Abrokwah*	2014	Abrokwah SO, Moser CM, Norton EC. The effect of social health insurance on prenatal care: the case of Ghana. Int J Health Care Finance Econ. 2014;14(4):385–406.	Prenatal care utilization & OOPE(s) per ANC visit	NR^1^	OOPE*** NHIS 3600GH¢ ($0.40 USD), uninsured 21,000 GH¢ ($2.40 USD) for the first ANC visit	NR^1^	NR^1^	Descriptive statistics, logistic regression	(7) Independent variables, dependent variable (prenatal OOPE)	1.) Insured women spend less on prenatal care compared to the uninsured. 2.) Having insurance increases the number of prenatal care visits by 24% relative to being uninsured.
*Abuosi*	2015	Abuosi A, Adzei F, Anarfi J, Badasu D, Atobrah D, Yawson A. Investigating parents/caregivers financial burden of care for children with non-communicable diseases in Ghana. BMC Pediatrics. 2015;15(1).	Financial burden/ OOPE direct inpatient care & perceived financial difficulties	NR^1^ used an arbitrary threshold of > 50 GH¢. as expensive or burdensome	NR^1^	NR^1^	NR^1^	Descriptive, logistic regression	(11) Independent variables, dependent variable (financial burden of care)	Uninsured respondents were twenty- three times more likely than the insured to make higher out of pocket payments for hospitalizations and more likely to experience financial burden of care.
*Kusi*	2015	Kusi A, Hansen K, Asante F, Enemark U. Does the National Health Insurance Scheme provide financial protection to households in Ghana? BMC Health Services Research. 2015;15(1).	Direct OOPE(s) for inpatient, outpatient care, & transportation cost	10% of total household expenditures & SE(s)^5^ at (20% & 40% thresholds)	OOPE*** OPD^3^; NHIS 6.7 GH¢ uninsured 25.5GH¢. IPD^4^*** NHIS 44.25GH¢ uninsured 86.73 GH¢. Transportation cost NS^2^	6% of NHIS respondents compared to 23.2% of the uninsured made CHE(s)	NR^1^	Descriptive statistics, logistic regression	(6) Independent variables, dependent variable (CHE)	1.) NHIS significantly reduces the probability of a household incurring CHE(s). 2.) Households with at least one member having a chronic illness were 94% higher than those without a chronic illness to incur CHE.
*Aryeetey*	2016	Aryeetey G, Westeneng J, Spaan E, Jehu-Appiah C, Agyepong I, Baltussen R. Can health insurance protect against out-of-pocket and catastrophic expenditures and also support poverty reduction? Evidence from Ghana’s National Health Insurance Scheme. International Journal for Equity in Health. 2016;15(1).	Direct OOPE(s) for inpatient, outpatient care, & transportation cost	SE(s)^5^ at (40% threshold)	IPD^4^ NS^2^ 2009 OOPE ***OPD^3^ NHIS GH¢ 19.8 uninsured GH¢ 27.4. 2011 OOPE*** OPD^3^ NHIS 26.1GH¢ uninsured 53.2GH¢. Transportation cost NR^1^	In 2009, 18.4% of NHIS respondents made CHE(s), compared to 36.1% uninsured. In 2011 7.1% NHIS & 28.7% Uninsured	NHIS households were 7.5% less likely to fall into poverty.	Descriptive statistics, logistic regression	(9) Independent variable Insurance status, dependent variable (OOPE)	1.) Enrolment in health insurance reduced household OOPE by 86%. 2.) Insured households were 3% less likely to make CHE(s). 3.) Being insured reduces households’ probability of falling into poverty by 7.5%.

*** Denotes statistically significant results ^1^NR Not reported by the studies. ^2^ NS Non-significant results

^3^OPD: Out-patient care ^4^ IPD: Inpatient care

^5^SE: Subsistence expenditures defined as non- food expenditures (typically set at 40% threshold for health expenditures exceeding this amount i.e. OOPE exceeding 40% of non-food expenditure is considered catastrophic)

The seven studies that met our inclusion criteria were published between 2008 and 2016 [26–32]. They were cross-sectional studies conducted across eight out of ten regions in Ghana. Six studies collected primary data in the form of a questionnaire administered to individuals or households. The seventh (Abrokwah 2014) analyzed secondary data, i.e., a subset of 1032 reproductive women from the Ghana Living Standard Survey-Round Five (GLS5), which is a standardized, nationally representative survey of approximately 18,000 households across all 10 regions in Ghana, conducted by the Ghana Statistical Service [29, 33]. It provides a comprehensive assessment of living conditions in Ghana, which includes the health status of the population, education, housing, income, consumption expenditure, access to financial services, and employment [33].

All seven studies asked participants to recall costs associated with seeking healthcare from two weeks to twelve months preceding the surveys. Respondents were caregivers of children, women of reproductive age, and all members of the households. The study populations ranged from 225 individuals to approximately 4000 households. (Abuosi 2015) addressed individuals who were parents/caregivers of children with NCDs and provided costs by insurance status but did not interview the children. All other studies were conducted on adult populations and sampled the entire household or provided secondary analysis of previously collected household data [30].

Apart from health care costs and insurance status, all studies report some information regarding the SES of the respondents, which invariably included education, income level, occupation, age, marital status, and employment status. However only four studies (Nguyen 2011, Abrokwah 2014, Dalaba 2014, and Kusi 2015) used a principal component analysis of ownership of assets, farmland, and household items in their household questionnaire to stratify the study population into socioeconomic wealth quintiles [27–29, 31]. This is the standardized method used by the Ghana Demographic Health Survey (DHS) as well as other DHS reports to measure inequalities in household characteristics, access to health services, and health outcomes [34].

All studies reported costs associated with seeking health care, which included direct medical costs, indirect costs such as transportation costs and lost productivity/wages. However, four studies (Nguyen 2011, Abrokwah 2013, Kusi 2015, Aryeetey 2016) reported significant differences in OOPEs between the insured and uninsured, and two studies (Chankova 2008 and Dalaba 2014) reported no difference [26–32]. One study (Abuosi 2015) did not examine differences in OOPE but reported on financial catastrophe [30]. Three studies reported differences in the cost of seeking care in both outpatient department (OPD) and inpatient department (IPD) by insurance status. Specifically, Aryeetey’s study was a survey of 3300 households in 2009 in the Central and Eastern regions of Ghana with a repeat survey of both regions in 2011 [32]. This study reported a baseline mean OOPE for OPD in 2009 of 19.8 GH¢ for the insured and 27.2 GH¢ for the uninsured. In the follow-up survey in 2011, OOPEs for OPD rose to 26.5 GH¢ for the insured and 53.5 GH¢ for the uninsured. The study found no significant difference in IPD OOPEs by insurance status for the two study periods [32]. In contrast, Kusi’s study of 2430 representative households across three ecological zones in Ghana did find significant differences in both IPD and OPD OOPEs by insurance status [31]. On average in this study, the uninsured paid 25 GH¢ for OPD and the insured paid 7 GH¢. For IPD, the uninsured households paid two times more than the insured (uninsured 86 GH¢, insured 44 GH¢), despite the fact that the insured were more likely to report an illness, and twice as likely to report a household member with a chronic medical condition, both of which were associated with higher OOPEs in the study.

Overall, the difference in magnitude of OOPs reported for all studies ranged from the uninsured paying between 1.4 to 10 times more than the insured for both inpatient and outpatient care.

In a study of 2500 households in two rural and poor districts in Ghana, (Nguyen 2011) found the uninsured spent two times more than the insured for surgical care and hospitalizations, and 1.5 times more for antenatal care and delivery [27]. The insured paid for services such as consultation fees, laboratory expenses, and drugs that were supposed to be covered under the insurance scheme. Interestingly, (Abrokwah 2013) in a secondary analysis of 1032 women of reproductive age from the GLS national survey, found that on average, insured pregnant women spent 3600 GH¢ ($0.40 USD) 95% CI (2700 GH¢ - 4900 GH¢) on their first antenatal visit and the uninsured spent 21,000 GH¢ ($2.40 USD) 95% CI (19,000 GH¢ - 23,900 GH¢) [29]. Insurance increased a women’s likelihood of seeking prenatal care, although the poorest women were still less likely to be insured [28].

Only three of the seven studies (Nguyen 2011, Kusi 2015, and Aryeetey 2016) reported financial catastrophe; one study (Aryeetey2016) also included differences in poverty reduction by insurance status [27, 31, 32].The measure of financial catastrophe most commonly used was subsistence expenditure, defined as household annual non-food expenditure with a 40% catastrophic threshold. Aryeetey’s study of 3300 households in 2009 found that 18% of insured households made catastrophic payments compared to 36% of uninsured households [32]. The proportions of individuals who made catastrophic payments declined in the 2011 follow-up survey, with only 7% of the insured incurring catastrophic payments compared to 29% of the uninsured. The authors also examined the impact of the NHIS on poverty reduction in the two study periods using mean monthly food expenditure. Households that spent less than the mean were considered poor [32]. The results showed that insured households were 7.5% less likely to fall into poverty. Kusi also reported that the insured were 4.2 times less likely to incur catastrophic payments compared to the uninsured. According to logistic regression analysis, household size, number of children under 5 years, ill health status of household members, female household head, and longer distance to the nearest health facility, in addition to insurance status, were statistically significant determinants of CHEs.

The three studies that reported financial catastrophe attempted to address some confounders in the relationship between insurance and CHE, i.e., perceived health status of the respondents, differences in utilization of health services, and household wealth characteristics, although the studies were limited by their retrospective design and the lack of matched controls. Nguyen examined catastrophic health payments at multiple income (5, 10%) and non-food expenditure (10 and 20%) thresholds and observed that NHIS coverage was associated with a reduced likelihood of incurring CHEs [27]. In a study of 225 caregivers of children with non-communicable diseases, Abuosi adopted an arbitrary amount of 50 GH¢ ($11 USD), over which health expenditures were considered financially burdensome or catastrophic [30]. Seventy percent of their study population paid 50 GH¢ or less, but 40% of them reported financial difficulties in caring for their children during the hospitalization for a non-communicable disease. In a logistic regression of socio-demographic factors, only insurance status and perceived financial difficulty were significant predictors of higher likelihood of experiencing financial burden or CHEs [30].

## Discussion

To our knowledge, this is the first systematic review on the impact Ghana’s NHIS on reducing out of pocket expenditures and catastrophic health payments. Although we identified only seven quality articles, findings from our review suggest that the NHIS has made some impact in reducing the financial burden of health care, which is consistent with evidence from other LMICs. Specifically, insurance has been associated with a 1 to 6% decrease in OOPEs in Indonesia (2%), Vietnam (6%), India (2%), Kenya (2%), Mali (3%), and Nigeria (3%) [35]. However, Mali, the Philippines, and China saw an initial 1 to 7% increase in OOPs with health insurance, which in China was attributed to increased utilization of health services by the insured and selection of higher level providers [35, 36]. Our results also show that although insured members paid less than the uninsured in OOPs, they were still at risk of facing catastrophic payments at the point of care. In the studies examined, between 6 to 18% of the insured made catastrophic payments, which is a significant problem as most of these studies were conducted in poor and economically-deprived regions in Ghana.

All studies reported the insured making OOPs at the point of care in the form of user fees, medicines, consultation fees, and informal fees; some included unofficial payment to providers. This remains a significant barrier to achieving UHC in Ghana and in many LMICs. In fact, medicines accounted for 40 to 60% of OOPEs in a study of 39 LMICs, and consistent with findings of our studies (Chankova, Kusi, Nguyen) that reported the cost of medicines [37]. The NHIS includes 522 medicines adopted from the WHO essential medicines list that are to be provided at no cost at the point of care [38]. Anecdotal reports of insured members having to purchase drugs privately due to lack of stock at facilities, as well as untimely and poor reimbursements of pharmacies by NHIS are amongst other reasons why the cost for medicines remains high and even catastrophic in some instances [39]. In fact, a study in rural Ghana found that spending on insulin for diabetes represented 60% of monthly income for individuals barely making minimum wage [40]. Similar observations have been made in Uganda, Mali, China, India, and Pakistan [41, 42]. When health care expenditures are compared across SSA, 80% of the countries continue to have CHEs, with OOPEs ranging between 20 to 70% of total health expenditures, thus indicating that health-care is still largely unaffordable across the region despite the advent of health insurance schemes [21].

Despite the strengths of our study, there are several limitations that can be addressed with future investigations on this topic. First, our comprehensive search identified only seven studies that adequately addressed our research questions, and only three were rated “high” in quality in reporting on the outcomes of OOPEs and CHEs. Furthermore, all studies used observational study designs and did not make comparisons with control groups (the uninsured) that were matched based on socio-demographic characteristics. This raises the issue of potential confounders in the relationship between insurance and health costs. In addition, we could not assess the methodological quality of the authors’ data collection process and how well information on insurance and cost was gathered. In some instances, insured members have to wait up to 3 months to use their cards and would essentially be considered uninsured and incur higher costs [32]. Furthermore, all costs incurred were self-reported and varied in terms of the recall period used in the studies. For these reasons, we could not perform a meta-analysis. Despite these limitations, as the first study to address this critical deficit in the literature, the results of this systematic review contribute to current knowledge of the impact of NHIS in financial risk protection in Ghana. Our review also emphasizes the need to develop guidelines and metrics to properly measure the impact of insurance on health costs. Directions for future investigations and policy recommendations are summarized in Table 5.

**Table 5:** Recommendations

Recommendations for future investigations	
• Improved study designs and metrics for measuring healthcare costs and expenditures	
• Controlling for confounders in the relationship between health insurance and out of pocket expenditures	
• Standardization of measures of affordability and house-hold capacity to pay using WHO methods	
• Further studies on how house-holds cope with making out-pocket payments for healthcare	
• Adjusting for inflation/deflation to allow for more time specific comparisons in order to provide better descriptions of acceptable health costs and living standards at any given time period	
Policy recommendations	
• Reducing the risk of OOPEs for medicines with a focus on improving the medical supply chain system	
• UHC policies need to clearly articulate the organization and standardization of health services that guarantee a minimum package.	
• Monitoring for effectiveness: Robust and sensitive indicators need to be collected routinely to inform timely interventions for the poor. This could include input costs of services, patient perspectives on quality of care, and human resource monitoring	

## Conclusion

This study—the first to systematically review the achievements of NHIS in providing financial risk protection in the last 14 years—shows that despite some impact in reducing the financial burden of care, health care expenditure remains catastrophic for many insured individuals in Ghana. Further investigations are needed to explore reasons why OOPEs persist, particularly for medicines, consultations, and laboratory tests that are included under the NHIS. Government-specific strategies to improve healthcare financing particularly for poor, at-risk populations is key to the sustaining NHIS’s mission to provide universal health coverage in Ghana.

## Additional file

Additional file 1: Search Strategy. The is a description of our comprehensive list of MeSH terms used to identify all studies on the impact of the national health insurance scheme of Ghana on out of pocket expenditures and financial catastrophe. (PDF 38 kb)

## Abbreviations

CHE: Catastrophic health expenditure
DHS: Demographic health survey
GLS: Ghana living standards survey
IPD: Inpatient department
LMIC: Low-and middle-income countries
MeSH: Medical subject headings
NHIS: National health insurance scheme
OOP: Out of pocket payment
OOPE: Out of pocket expenditures
OPD: Out-patient department
PICO: Population, intervention, comparison, outcome framework for evidence based practice
PRISMA: Preferred reporting items for systematic reviews and meta-analysis
SES: Socio-economic status
SSA: Sub-Saharan Africa
WHO: World health organization
