# Supplementary material for: Evaluating the impact of the national health insurance scheme of Ghana on out of pocket expenditures: a systematic review
Source: BMC Health Serv Res. 2018 Jun 7;18:426. doi: 10.1186/s12913-018-3249-9 (PMC5992790; doi:10.1186/s12913-018-3249-9)
Supplement: Supplementary file 1 — Search Strategy. The is a description of our comprehensive list of MeSH terms used to identify all studies on the impact of the national health insurance scheme of Ghana on out of pocket expenditures and financial catastrophe. (PDF 38 kb) [file 12913_2018_3249_MOESM1_ESM.pdf]

Search strategy

Combined PubMed MeSH Terms				
**Insurance**			**Country**	**English**
Insurance<br>*National health program(s)*<br>*Insured*<br>*Uninsured*<br>*National health*<br>*Catastrophic health*<br>*Universal health*<br>*Universal coverage*<br>*Health coverage*	AND	*Ghana*<br><br><br>*Ghanaian*	AND	
